# Bis(triethoxysilyl)ethane (BTESE)–Organosilica Membranes for H_2_O/DMF Separation in Reverse Osmosis (RO): Evaluation and Correlation of Subnanopores via Nanopermporometry (NPP), Modified Gas Translation (mGT) and RO Performance

**DOI:** 10.3390/membranes14010008

**Published:** 2023-12-26

**Authors:** Suhaina Mohd Ibrahim, Ken-ichi Sawamura, Kengo Mishina, Xin Yu, Feridoun Salak, Shigeru Miyata, Norihiro Moriyama, Hiroki Nagasawa, Masakoto Kanezashi, Toshinori Tsuru

**Affiliations:** 1 eSep Inc., Keihanna Open Innovation Center @ Kyoto (KICK), Annex 320, 7-5-1 Seikadai, Seika-cho, Soraku-gun, Kyoto 619-0238, Japan; mishina@esep.co.jp (K.M.); yu_xin@esep.co.jp (X.Y.); miyata@esep.co.jp (S.M.); 2 Department of Chemical Engineering, Hiroshima University, 1-4-1 Kagamiyama, Higashihiroshima 739-8527, Japan; moriyama-n@hiroshima-u.ac.jp (N.M.); nagasawa@hiroshima-u.ac.jp (H.N.); kanezashi@hiroshima-u.ac.jp (M.K.); tsuru@hiroshima-u.ac.jp (T.T.)

**Keywords:** N,N-dimethylformamide, organic solvent reverse osmosis, organosilica membranes

## Abstract

A 40 cm length Bis(triethoxysilyl)ethane (BTESE) membrane having different pore sizes was successfully prepared by changing the number of coating times for gas permeation (GP) and organic solvent reverse osmosis (OSRO) separation study. It was found that BTESE-6 membranes prepared through six-time coating consisted of small-sized pores in the range 0.56 to 0.64 nm estimated using modified Gas Translation (mGT) method and 0.59 to 0.67 nm estimated by nanopermporometry (NPP) method, respectively. These membranes demonstrated a high DMF rejection, *R*_DMF_ > 95% with total flux, *J*_v total_ > 5 kg m^−2^ h^−1^ at operating condition feed pressure, *P*_f_: 8 MPa; feed temperature, *T*_f_: 50 °C; and feed flowrate, *Q*_f_: 30 mL/min; and they exhibited a high degree selectivity of He/SF_6_ in the range of ~ 260–3400 at a permeation temperature 200 °C. On the other hand, the larger pore sizes of the BTESE-4 membranes (pore size estimates > 0.76 nm to 1.02 nm) exhibited low DMF rejection and a low degree selectivity of He/SF_6_ around ~30% and 25, respectively, at the same operating condition as BTESE-6. Both GT and NPP methods can be considered as an indicator of the measurement membrane pore size. From this study, it was found that He and SF_6_ gases can be some of the potential predictors for water and DMF permeance. Furthermore, by comparing our OSRO membrane with other PV membranes for DMF/H_2_O separation, our BTESE-6 membranes still exhibited high flux in the range of 3–6 kg m^−2^ h^−1^ with a separation factor H_2_O/DMF in the range of 80–120.

## 1. Introduction

N,N-dimethylformamide (DMF) is a polar solvent with a high boiling point (153 °C) that can dissolve both in water and most organic solvents. It is commonly used for extracting acetylene and manufacturing polyacrylonitrile fibers, as well as in organic synthesis, dyes, pharmaceuticals, and petroleum refining and resin industries [1,2,3]. After being used, the DMF concentration up to ~5–25 (wt.%) in wastewater is difficult to be recycled with a distillation process and, moreover, it is known that DMF is chemically stable in wastewater and can hardly biodegrade [3,4]. Hence, it is necessary to recover the DMF from wastewater from an environment perspective.

Many inorganic nanoporous membranes such as zeolites [5,6,7,8] and organically bridged silicas [9,10,11,12] have been studied both theoretically and experimentally for their ability to reject ions in reverse osmosis for the purpose of separating salt from aqueous solutions. Previous research by Hiroshima University has shown that a bis(triethoxysilyl)ethane (BTESE) can withstand temperatures up to 80 °C and also exhibit excellent chlorine resistance in desalination applications with no significant changes in filtration performance [11,13]. Additionally, BTESE membrane was found to remain stable in performance even after fouling studies using four different types of foulants, including bovine serum albumin (BSA), sodium alginate (SA), sodium dodecyl sulfate (SDS), and dodecyl trimethyl ammonium bromide (DTAB) surfactants [13]. These four foulants are typical effluent organic matter (EOM) and industrial waste. Based on this finding, it motivates us to apply the RO BTESE membrane to H_2_O/DMF separation.

Normally, RO membrane pore size is below 0.5 nm [14]. The molecular separation in BTESE membranes is usually achieved through either pore-size-based molecular sieving or affinity-based separation. The preparation of such membranes with pore sizes <1 nm is highly suitable through sol–gel technology, which involves the hydrolysis and polycondensation of silicon alkoxides to form a cross-linked silica network [15]. This method enables the production of materials with the desired morphological characteristics by adjusting the types and amounts of precursors and solvents [16,17,18] as well as the concentrations of water [19,20,21,22,23] and acid [17,22,23]. Hiroshima University’s successful strategy for controlling the water ratio (WR) to design the pore networks of BTESE membranes for both gas separation and reverse osmosis applications has been demonstrated through studies [11,13,20]. Additionally, there are many who conducted research on multiple generations of organoalkoxysilanes, such as bis(triethoxysilyl)methane (BTESM) and 1,3-bis(triethoxysilyl)propane (BTESP), 1,4-bis[2-(triethoxysilyl)vinyl]benzene (BTES-VB), and 2,5-bis[2-(triethoxysilyl)vinyl]pyridine (BTESVP) to tailor the pore size of organosilica membranes for gas separation (GS), pervaporation (PV), and RO applications [9,10,11,12,13,20,24,25,26,27,28,29,30].

Therefore, in order to modify the pore size distribution in our BTESE membranes for GS and RO experiments, we altered the number of coating layers in the current work. Using the Gas Translation (GT) model and nanopermporometry (NPP) techniques, the pore size distribution of these membranes was assessed. Additionally, this study reports the relationship between gas and water permeance. We compared the performance of our membranes with other PV membranes because, as far as we are aware, there has only been a small amount of research published on the BTESE membranes in the H_2_O/DMF separation by employing RO membranes [31,32,33,34].

## 2. Materials and Methods

### 2.1. Preparation of BTESE-Derived Sols and Membranes

An ethanol (EtOH) solution was used to uniformly dissolve BTESE, an organosilica precursor. The solution was then vigorously stirred while a mixture of H_2_O and nitric acid (HNO_3_) was added dropwise. The end solution had the molar ratios of BTESE/H_2_O/HNO_3_ = 1/x/0.2 (x = 240). By varying the amount of EtOH added to the solutions, the concentration of BTESE was kept constant at 5 wt.%. After that, the mixture was continuously stirred in a closed system at 25 °C for six hours to enable the development of silica sols and was aged for 8 days at a temperature of 50 °C before it could be used as a top layer.

The BTESE-derived silica membranes were supported by porous α-alumina tubes with an outer diameter of 10 mm, 50% porosity, and an average pore size of 3 μm. Initially, the outer surface of a porous support was coated with α-alumina particles, with an average particle diameter of 0.2 μm. The surface was then fired for 10 min at 550 °C to create an intermediate layer of α-alumina. Ultimately, the BTESE-derived organosilica top layer was created by coating a BTESE solution, drying it, and firing it for 30 min at 300 °C under air. A detailed explanation of the membrane fabrication can be found in our previous manuscript [35].

The membranes were prepared in different pore sizes to study the pore size distribution effect on the DMF/H_2_O RO separation performance. The different pore sizes were attributed to the different numbers of the coating layers of the BTESE solution on the membrane support. BTESE-4, BTESE-5 and BTESE-6 denote the 4, 5 and 6 coating times of BTESE solution, respectively. Multiple membranes were prepared for each coating time and are referred to as BTESE-x-x. The first and the last digit indicate membrane type (coating times) and the serial number, respectively. There are 2, 2 and 5 membranes that were used for coating times of 4, 5, and 6, respectively. Table 1 lists all the membranes that were used in this study.

### 2.2. Membrane Pore Size Distribution

By employing water (H_2_O) as a condensable vapor in a nanopermporometry (NPP) technique, the pore size distribution (PSD) of the membranes was estimated. The measurement of pore size distributions between 0.5 and 50 nm was suggested for this approach. In this work, the permeance of nitrogen in a mixture of nitrogen and water at 25 °C was measured as a function of the water vapor pressure.

### 2.3. Gas Permeation (GP) and Reverse Osmosis (RO) Performance

Prior to the RO measurement, the GP measurement was carried out at 200 C using single components of He, H_2_, N_2_, CH_4_, CF_4_, and SF_6_. A single gas, with a transmembrane pressure of 0.04–0.1 MPa, was supplied to the membrane module and the permeate stream was maintained at atmospheric pressure. Equation (1) gives the gas permeance, *P* (mol m^−2^ s^−1^ Pa^−1^).

(1) P=n/AΔp

In Equation (1), *n* is the permeate flow rate (mol s^−1^). The transmembrane pressure difference (Pa) and membrane surface area (m^2^) are denoted by *A* and Δ*p*, respectively. Equation (2) then defines *α_A/B_* (-), the ideal selectivity for gas *A* over gas *B*, as the ratio of their gas permeance.

(2) $$\alpha_{A/B}=P_{A}/P_{B}$$

**Figure 1:**
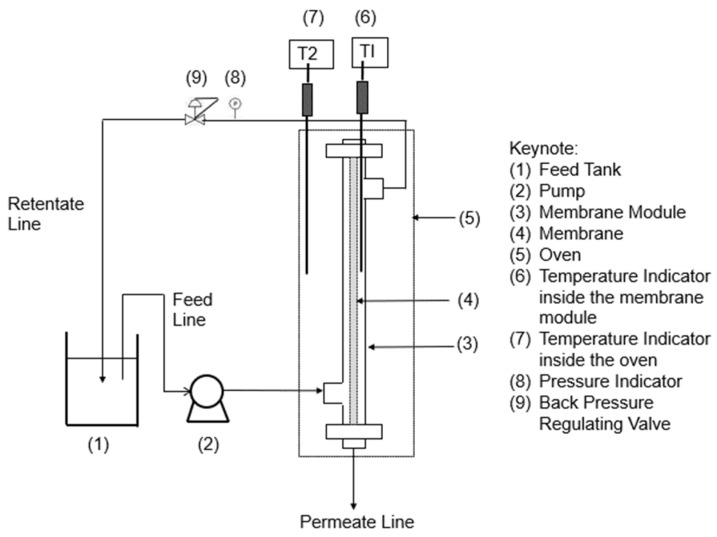
Schematic diagram of the experimental apparatus for reverse osmosis (RO) measurement.

The RO experiment was conducted as illustrated in Figure 1. A plunger pump (dual pump KP-21 series; FLOM Co., Tokyo, Japan) was used to supply the 6 wt.% DMF aqueous solution to the shell side of the BTESE tubular membrane at a flow rate of 30 mL/min. The shell side was also subjected to a pressure of 4–8 MPa. Unless otherwise noted, the oven’s temperature was kept between 25 and 50 °C. Every hour, the mass of the solution that entered the bore side from the shell side was measured. Atmospheric pressure was maintained in the permeate stream. The feed container was filled with the recycled retentate. A refractometer (RX-5000i) (Atago, Tokyo, Japan) was used to measure the concentrations of feed, retentate, and permeate.

The experimental data could be used directly to calculate solutes’ rejection, *R*_solutes_ (*R*_DMF_), and total flux, *J*_w,total_.

Solutes’ rejection, *R*_solutes_ (%), was expressed as follows (Equation (3)):

(3) $$R_{\mathrm{solutes}}=(1-w_{p}/w_{f})\times 100$$

where the DMF concentrations of feed and permeate are expressed with mass fraction, $w_{f}$ and $w_{p}$ (wt.%), respectively. Meanwhile, the following formula (Equation (4)) was used to determine the total flux, *J*_w,total_ (kg m^−2^ h^−1^):

*J*_w,total_ = *W*_p_/(*tA*) (4)

where *A* is the membrane area (m^2^), *t* is the time for collecting the permeate (h), and $W_{p}$ is the mass of the permeate solutions (kg). Equation (5) below was used to calculate the water (H_2_O) flux, $J_{w,\;H_{2}O}$ (kg m^−2^ h^−1^) using the H_2_O mass fraction in the permeate line, $w_{,\;H_{2}O_{p}}$(-):

(5) $$J_{w,H_{2}O}=J_{w,\mathrm{total}}.w_{,H_{2}O_{p}}$$

The water permeability, *L*_p_, was calculated using Equation (6):

(6) $$L_{p,H_{2}O}\;J_{w,H_{2}O}/(\Delta p-\Delta \pi )$$

where Δ*p* and Δπ are the differences in applied pressure and osmotic pressure, respectively.

The solute flux, *J*_s_ (kg m^−2^ h^−1^) and solute permeability, *P_s_* (kg m^−2^ h^−1^), through the membrane were calculated using Equations (7) and (8) below.

(7) $$J_{s}=J_{w,\mathrm{total}}.w_{,DMF\;p}\;$$

(8) $$J_{s}=P_{s}(w_{,DMF\;f}-w_{,DMF\;p})$$

where ($w_{,DMF\;f}$ − $w_{,DMF\;p}$) is the concentration difference between feed and permeate.

The osmotic pressure, π, is defined based on the activity, *a* (=γx), as shown in Equation (9). In Equation (9), R is the gas constant, 8.314 J mol^−1^.

K^−1^; T is operation temperature, 25 °C, K; ν is the molar volume, m^3^ mol^−1^; r is the activity coefficient which can be calculated by Wilson equation; and x is the molar fraction of each component.

(9) $$\pi =-\frac{RT}{\nu }\ln a=-\frac{RT}{\nu }\ln\left(\gamma x\right)$$

For every experimental data point presented in this manuscript, the average value of three samplings is indicated. The experimental error during each measurement was less than 5%.

## 3. Results and Discussions

### 3.1. Gas Permeation (GP) Performance of BTESE Organosilica Membranes

**Figure 2:**
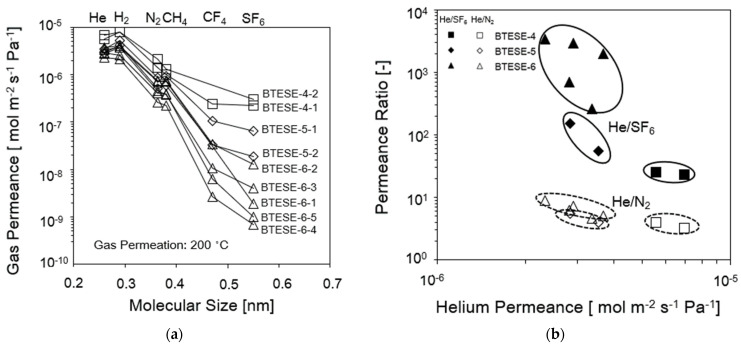
(**a**) Single gas permeance vs. molecular size. (**b**) Permeance ratio He/N_2_ and He/SF_6_ vs. He permeance of the BTESE organosilica membranes fired at 300 °C under air at a permeation temperature of 200 °C.

Gas permeance was measured using He, H_2_, N_2_, CH_6_, CH_4_, CF_4_, and SF_6_ at a permeation temperature of 200 °C in order to examine the permeation efficiency of BTESE membranes that were calcined at 300 °C, as shown in Figure 2a. All the membranes chosen and used for this study consist of membranes having varieties of permeances and selectivities as the main purpose of this study is to clarify the effect of pore size on the membrane performance. The gas permeances of these 2 BTESE-4, 2 BTESE-5 and 5 BTESE-6 membranes are plotted as a function of molecular size, and it is noteworthy that all the 3 types of BTESE membranes exhibited a He permeance higher than 10^−6^ mol m^−2^ s^−1^ Pa^−1^ despite difference in the pore sizes. BTESE-4-1 and BTESE-4-2 membranes showed a noticeable higher H_2_ permeance than that of He, which suggests the Knudsen mechanism is dominant for the permeation of He and H_2_ through the loose amorphous networks in a BTESE-4 membranes compared to BTESE-5 and BTESE-6 membranes. The same phenomenon was observed by Lee et al. [29].

However, Figure 2b demonstrates that all these three types of BTESE membranes have moderate He/N_2_ selectivity of approximately 3 to 9 but a high and wide range of the He/SF_6_ selectivity around 23–3405. The He/SF_6_ selectivity for the BTESE-4 membrane was approximately 23–25, while the He/SF_6_ selectivity increased drastically (to 260–3406) for BTESE-6 due to the smaller pore size network. Figure 2b also shows that we are able to reasonably control the pore sizes of BTESE derived membranes via coating times. BTESE-6-type membranes show scattered SF_6_ permeances, but similar permeance for small molecules such as He and N_2_ due to a small number of large pores. Hence, it shows that an increase in the number of the coating layers can improve the membrane performance. The detailed information of these gas separation properties is shown in Table 2.

### 3.2. Evaluation of Pore Size BTESE Membranes

In order to determine membrane pore sizes of less than 1 nm, the original GT model presented by Xiao and Wei [36] and Shelekhin et al. [37] was modified to create the mGT model. Using a modified Gas Translation (mGT) model method, we were able to quantify the pore size of these membranes [29]. Gas permeance through a microporous membrane under the mGT model can be expressed as in Equation (10).

(10) $$P_{i}=\frac{k_{0i}}{\sqrt{M_{i}\;RT}}\exp\left(\frac{E_{p,i}}{RT}\right)$$

where *P*_*i*_, *M*_*i*_, *R*, *T*, and *E*_*p,i*_ are the permeance, molecular weight, gas constant, temperature, and activation energy for permeation, respectively. $k_{0i}$ is a constant that depends on only membrane structures and is independent of the permeating gas species, as shown in Equation (11) below:

(11) $$k_{0i}=\frac{\varepsilon_{i}d_{0}\rho_{g,i}}{\tau_{i}L_{i}}\sqrt{\frac{8}{\pi }}$$

where $\varepsilon_{i},\;\tau_{i},\;L_{i}\;\mathrm{and}\;d_{0}$ are the porosity, tortuosity, thickness and pore size of the membrane.

Meanwhile the *ρ*_*g,i*_, can be defined as the ratio of the effective cross-sectional area of a pore for *i* component, *A_i_* to the physical cross-sectional pore area of the pore *A*_0_ and the random factor 1/3 represent for the three-dimensional space as indicated in Equation (12).

(12) $$\rho_{g,i}=\frac{1}{3}\frac{A_{i}}{A_{0}}$$

The mGT model assumes no structural size of permeating molecules [38]. As a result, *k*_0_ is independent of any permeating molecules and the *k*_0,*i*_ of a large gas such as SF_6_ is exactly the same as a small gas such as He. Since, the center of the *i*-th component cannot approach the wall, the diffusion distance in Equation (11) is assumed to be (*d*_0_ − *d_i_*) instead of *d*_0_. Therefore, the area of the pore opening effective for diffusion, *A_i_*, is proportional to the effective pore area Equation (13), which leads to the mGT model as in Equation (14)

(13) $$\rho_{g,i}=\frac{1}{3}\frac{A_{i}}{A_{0}}=\frac{1}{3}\frac{\pi {\left(d_{0}-d_{i}\right)}^{2}/4}{\pi d_{0}^{2}/4}=\frac{1}{3}\frac{{\left(d_{0}-d_{i}\right)}^{2}}{d_{0}^{2}}$$

(14) $$P_{i}=\frac{\varepsilon_{i}}{3\tau_{i}L_{i}}\left(d_{0}-d_{i}\right)\frac{{\left(d_{0}-d_{i}\right)}^{2}}{d_{0}^{2}}\sqrt{\frac{8}{\pi M_{i}RT}}\exp\left(\frac{E_{p,i}}{RT}\right)=\frac{k_{0}}{\sqrt{M_{i}RT}}{\left(d_{0}-d_{i}\right)}^{3}\exp\left(-\frac{E_{p,i}}{RT}\right)$$

In Equation (14), *k*_0_ is a constant that depends on only membrane structure and is independent of the permeating gas species. When *E_p,i_* is a constant, the pore size *d*_0_ can be obtained by regressing each ${\left(\sqrt{M_{i}\;}\;P_{i}\right)}^{\frac{1}{3}}$ to Equation (15).

(15) $${\left(\sqrt{M_{i}\;}\;P_{i}\right)}^{\frac{1}{3}}={\left(\frac{k_{0}}{\sqrt{RT}}\exp\left(-\frac{E_{p,i}}{RT}\right)\right)}^{\frac{1}{3}}\left(d_{0}-d_{i}\right)$$

**Figure 3:**
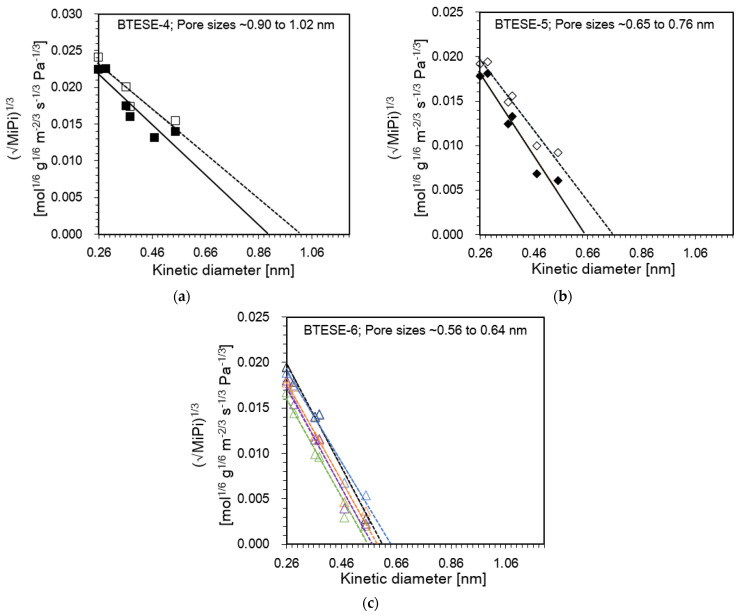
The pore size estimation via the mGT model as a function of kinetic diameter of permeating gases for (**a**) BTESE-4; (**b**) BTESE-5 and (**c**) BTESE-6 (symbols and lines indicate the experimental data and the fitting lines, respectively).

Figure 3a–c shows the the correlation between MiPi13 and the molecular size of permeated gases of 2 BTESE-4, 2 BTESE-5 and 5 BTESE-6 membranes. The estimated membrane pore size as expressed as the intercept of the *x*-axis MiPi13=0. It seems the estimated pore size of BTESE membranes shifted from a large (BTESE-4) to a small (BTESE-6) size, i.e., from 1 to 0.56 nm. This was because the BTESE-6 membranes consisted of a smaller pore network compared with the BTESE-4 membranes. As another possible reason, the defect holes in the surface of BTESE-6 membranes may be reduced due to an increase in the coating layers, which helps to reduce the unselective permeability from pinholes. Hence, as a result shown in Table 2, mostly the BTESE-6 membranes exhibit higher selectivity with lower gas permeances compared BTESE-4 and BTESE-5 membranes.

However, the nanopermporometry (NPP) method, which measures the rate at which non-condensable gas permeates a porous membrane after a mixture of condensable vapor and non-condensable gas is fed to the membrane, can also be used to determine the sizes of the pores [39,40,41]. The well-known Kelvin Equation states that vapor condenses at vapor pressure, *P*, even lower than the saturated vapor pressure, *P_s_*, in a capillary with a smaller pore size (radius *r_p_*) (16)

(16) $$RT\ln\left(\frac{P}{P_{s}}\right)=2\nu \;\frac{\sigma cos\theta }{r_{p}}$$

where *v*, *σ* and *θ* are molar volume, surface tension and contact angle, respectively. It is proven that capillary condensation occurs in a smaller pore at a lower relative pressure of vapor, i.e., *P*/*P_s_*. By determining the permeation rate as a function of the vapor pressure of condensable gas in the feed stream, one can estimate the pore size distribution because it can be assumed that the condensed vapor prevents non-condensable gas from penetrating. Put another way, a higher vapor pressure is needed to prevent a non-condensable gas from penetrating large pores.

**Figure 4:**
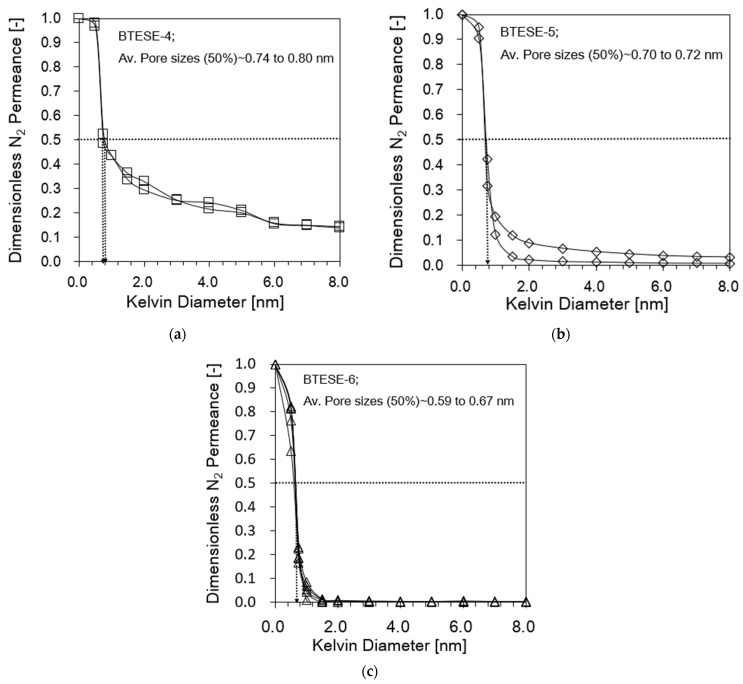
Dimensionless N_2_ permeance of BTESE organosilica membranes fired at 300 °C under air as a function of Kelvin diameter as determined by nanopermporometry (av. pore size 50%) (condensable vapor/water): (**a**) BTESE-4; (**b**) BTESE-5 and (**c**) BTESE-6.

Figure 4 shows the pore size distribution (PSD) curves of various BTESE membrane types as determined by NPP (using dry nitrogen as a non-condensable gas and water as a condensable vapor). The DP (dimensionless permeance) of nitrogen was plotted against Kelvin diameter. The contact angle is taken to be zero in this instance. As the Kelvin diameter increased, the DP value decreased. Using the interpolation of the permeance curve at a 50% relative permeance, the nominal pore radius was determined. The approximate pore sizes of the BTESE-4, BTESE-5, and BTESE-6 membranes used in this investigation are 0.59 and 0.67 nm, 0.70 to 0.72 nm, and 0.74 to 0.80 nm, respectively.

**Figure 5:**
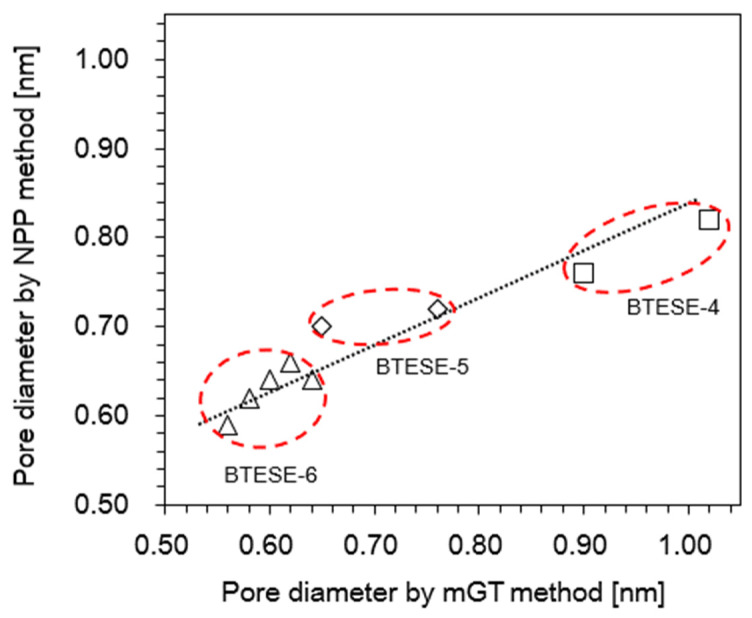
Correlation between pore size estimation for BTESE membranes fired under air using NPP versus mGT methods.

Since the pore sizes were calculated using two different methods, mGT and nanopermporometry, we tried to plot the correlation between these two methods. It should be noted that only pores effective (active pores) were taken into account and the dead-end pores, which made no contribution to permeation were not used for the pore size evaluation since both methods are based on permeation properties. Figure 5 illustrates pore sizes from these two approaches predicted were roughly comparable. This suggests that the pore sizes determined by the two methods are useful predictors of the membrane pore size. As far as we are aware, there is no publication reported on the correlation between NPP and mGT models yet. Additionally, this outcome demonstrated the stability of the BTESE membranes in both the gas phase and the gas-vapor phase.

### 3.3. Reverse Osmosis (RO) Performance of BTESE Organosilica Membranes

#### 3.3.1. Mechanical and Hydrothermal Dependency on the BTESE Membrane for DMF Separation

**Figure 6:**
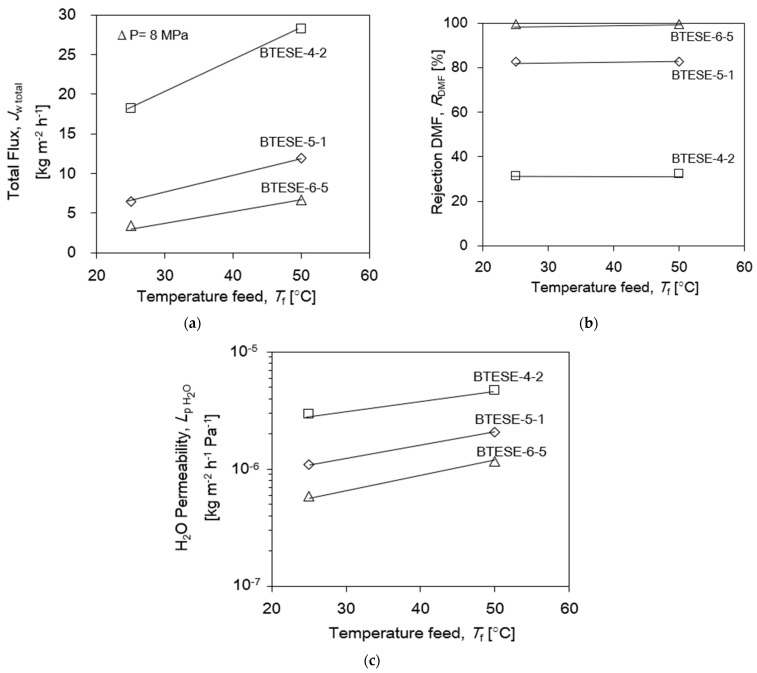
(**a**) Total Flux, *J*_w,total_. (**b**) DMF rejection, *R*_DMF_. (**c**) Water permeability, *L*_p,H2O_ versus temperature feed at operating conditions *P*_f_~8 MPa, *T*_f_~25–50 °C, and 6 wt.% DMF.

The influence of the temperature-induced RO performance of BTESE membranes is shown in Figure 6. BTESE-4-2, BTESE-5-1 and BTESE-6-5 membranes were chosen to represent membranes of each coating times group. It should be noted that, in Figure 6a,b, the total flux *J*_w,total_ increased as the operating temperature increased from 25 to 50 °C without diminishing the rejection of DMF (*R*_DMF_), which suggests that the pore sizes of these membranes were not influenced by the thermal expansion in the present operating temperature range. The water permeation performance of the membranes as a function of temperature is shown in Figure 6c. The water permeance, *L*_p,H2O_, also increased as the operating temperature increased from 25 to 50 °C, indicating an activated diffusion where permeances increase with temperature. An activated transport mechanism, that is commonly observed with the microporous materials, may be responsible for the transport through the BTESE membranes.

**Figure 7:**
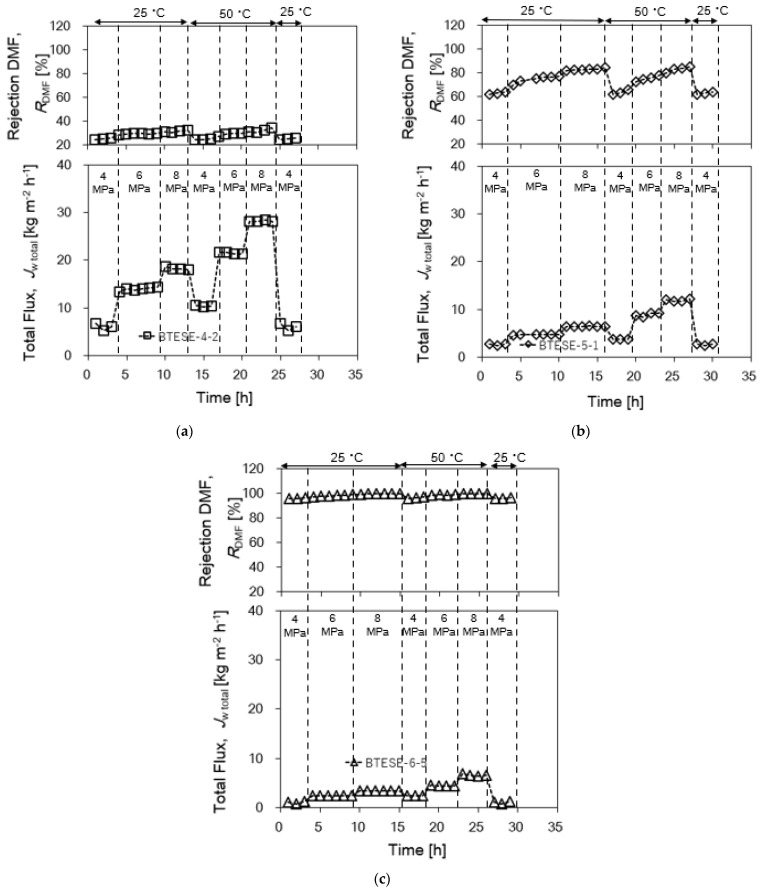
(**a**) BTESE-4-2, (**b**) BTESE-5-1 and (**c**) BTESE-6-5 at operating conditions *P*_f_~4–8 MPa, *T*_f_~25–50 °C, and 6 wt.% DMF.

As seen in Figure 7a–c, BTESE-4-2, BTESE-5-1 and BTESE-6-5 membranes were chosen to represent each coating times groups and tested for more 25 h at temperatures between 25 and 50 °C and *P*_f_ between 4 and 8 MPa with 6wt.% DMF feed concentration in order to examine the mechanical and hydrothermal durability of the membranes. At a pressure of 8 MPa and a temperature of 50 °C, the total flux for all three membranes was greater than 5 kg m^−2^ h^−1^. These findings also imply that at high pressure and different temperatures, there was no change in the effective pore diameters and structures as the *R*_DMF_ remained the same. It should be noted that at the end of the time course (Figure 7a–c), RO performances of all BTESE membranes which have evaluated under the same condition of the initial RO experiment showed approximately the same *R*_DMF_ and *J*_w_, confirming the stable and reproducible RO performance. The primary component of the BTESE structure, which is made up of chemically stable bonds like Si-C and Si-O-Si, is responsible for the consistent thermal stability found in the data study.

#### 3.3.2. Pressure Dependency of the BTESE Membrane for DMF Separation

**Figure 8:**
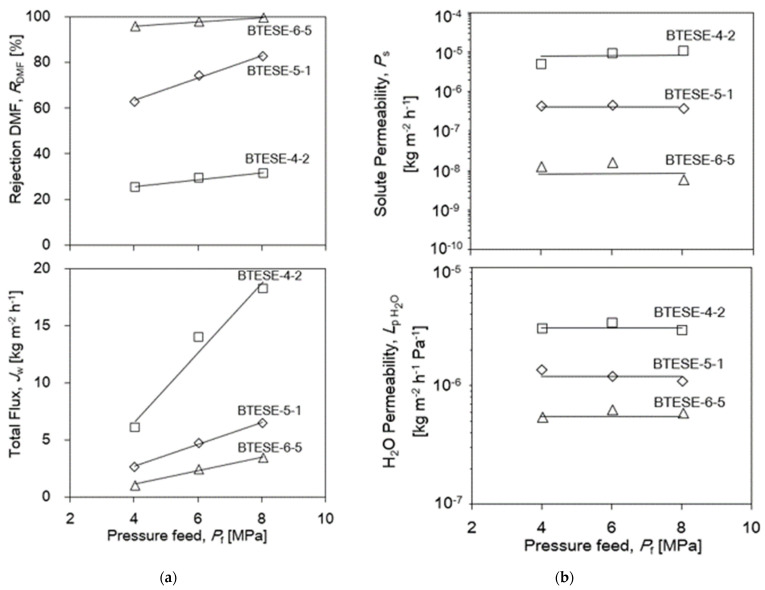
(**a**) Rejection DMF, *R*_DMF_ and Total Flux, *J*_w,total_. (**b**) Solute permeability, *P_s_* and H_2_O Permeability for BTESE organosilica membranes fired at 300 °C under air as a function: *P*_f_: 4–8 MPa; at *T*_f_: 25 °C; *C*_DMF_: 6wt.% DMF; *Q*_f_: 30 mL/min.

Again, BTESE-4-2, BTESE-5-1 and BTESE-6-5 membranes are used to illustrate how feed pressure affects permeation flux and DMF rejection in Figure 8a. As operating pressure increased, the total flux (*J*_w,total_) for all three membranes increased concurrently. Rejection is higher for the tighter membrane, as would be expected. All of the membranes reject the solutes in the following order: BTESE-6-5>BTESE-5-1>BTESE-4-2. The formation of small pore sizes in the BTESE-6-5 membrane resulted in a higher rejection but a lower water flux. Reverse osmosis membranes typically exhibit an increase in rejection as operating pressure increases, depending on solute permeability and permeate flux. According to Equations (6) and (8), the water (H_2_O) permeability, *L*_p,H2O_, and solute permeability, *P_s_*, in Figure 8b were nearly constant with pressure at 25 °C. This is in line with the fundamentals of the solution–diffusion (SD) model [42]. The water transport across the membrane is driven by the transmembrane pressure difference (ΔP-Δπ). Hence, the liquid-phase state of water permeation through BTESE membrane subnanopores was confirmed.

### 3.4. Relationship of Reverse Osmosis (RO) Performance with the Gas Permeation (GP) and Pore Size

**Figure 9:**
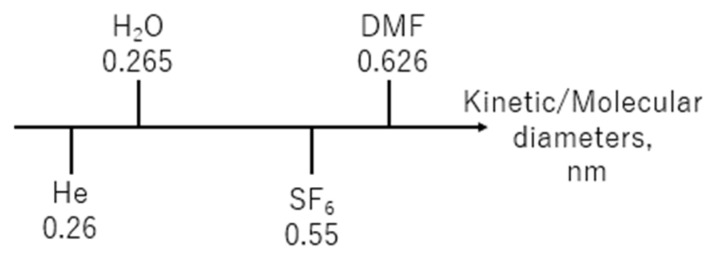
Schematic of the sequence order of these all solutes, liquids and gases.

As previously mentioned, it is recognized that a molecular sieving phenomenon which is generally accepted for single-gas permeation influences the transport mechanism of water and DMF during RO. For a BTESE membrane to be more appropriate for molecular sieving, its pore size needs to be between that of the solute and water. According to Sirkar et al. [1], assuming a spherical molecule, the molecular diameter of DMF is approximately 0.626 nm. Conversely, the He and SF_6_ gases have molecular diameters of 0.26 and 0.55 nm, respectively. A schematic of the order in which these solutes, liquids, and gases are displayed is shown in Figure 9. As a result, it makes sense to use SF_6_ gas to predict DMF permeance and He gas to predict water permeance.

**Figure 10:**
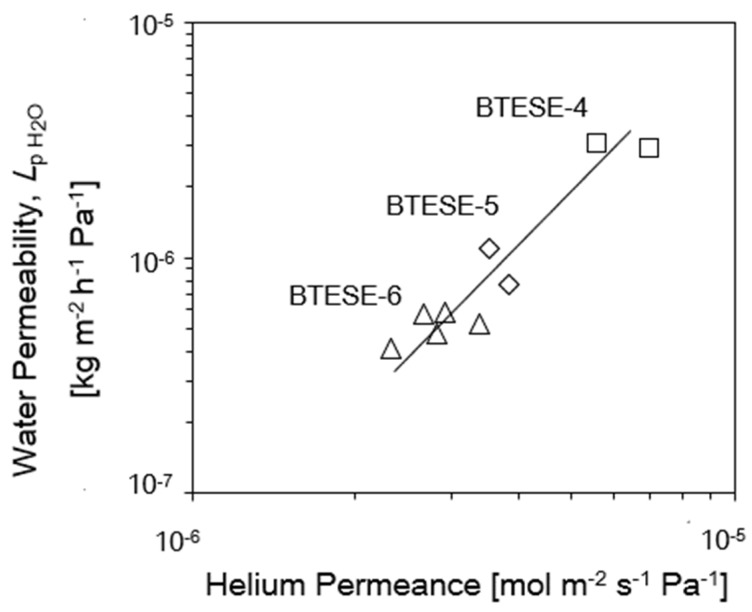
Water permeability, *L*_p,H2O_ (*P*_f_ = 8 MPa; *T*_f_ = 25 °C) versus Helium permeance (at *T*_f_ = 200 °C) for BTESE membranes.

As shown in Figure 10, the water permeability, *L*_p,H2O_, was plotted against He permeance. Note that the BTESE-4, BTESE-5, and BTESE-6 membrane performances are indicated by each point. For BTESE membranes, *L*_p,H2O_ increased as He permeance increased. Large-pore BTESE-4 membranes showed high *L*_p,H2O_ values and He permeance, whereas BTESE-6 membranes demonstrate the opposite. This result validates the consistent correlation between RO performance and pore size estimation using mGT and NPP methods.

To determine whether there might be a relationship between DMF rejection and gas permeation, the rejection of DMF was plotted against the He/SF_6_ permeance ratio. In this study, the DMF rejection and the He/SF_6_ permeance ratio were measured at 25 °C and 200 °C, respectively. The BTESE membrane’s pore size distribution can be measured using the permeance ratio He/SF_6_, where we can observe that the smaller the pore sizes will exhibit the higher, He/SF_6_ permeance ratios.

**Figure 11:**
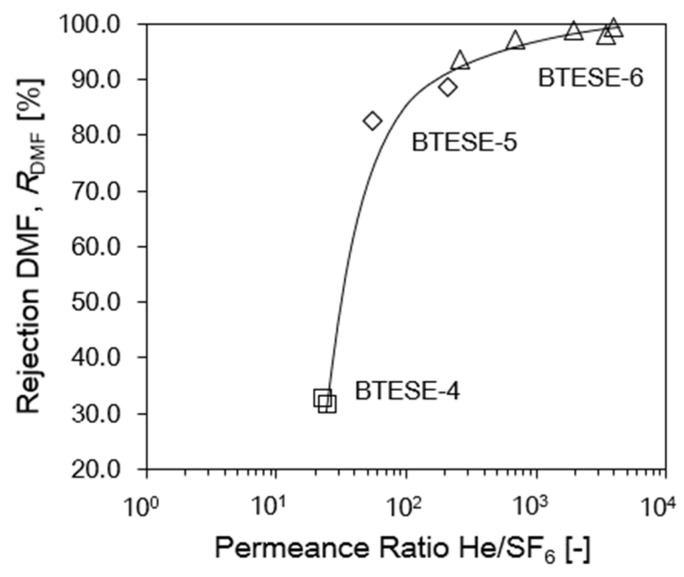
Rejection DMF, *R*_DMF_ (*P*_f_ = 8 MPa; *T*_f_ = 25 °C) versus Permeance Ratio He/SF_6_ (at *T*_f_ = 200 °C) for BTESE membranes.

As seen in Figure 11, the rejection of DMF increased along with an increase in the permeance ratio of He/SF_6_. Large pore size membranes were plotted at the bottom left of Figure 10 due to poor separation performance, while membranes with small pore sizes (BTESE-6) are plotted at the top right of the figures because these membranes (BTESE-4) possessed high separation performance. These findings indicate that it is reasonable to use SF_6_ as a predictor of DMF penetration and He gas as a predictor of water permeance.

**Figure 12:**
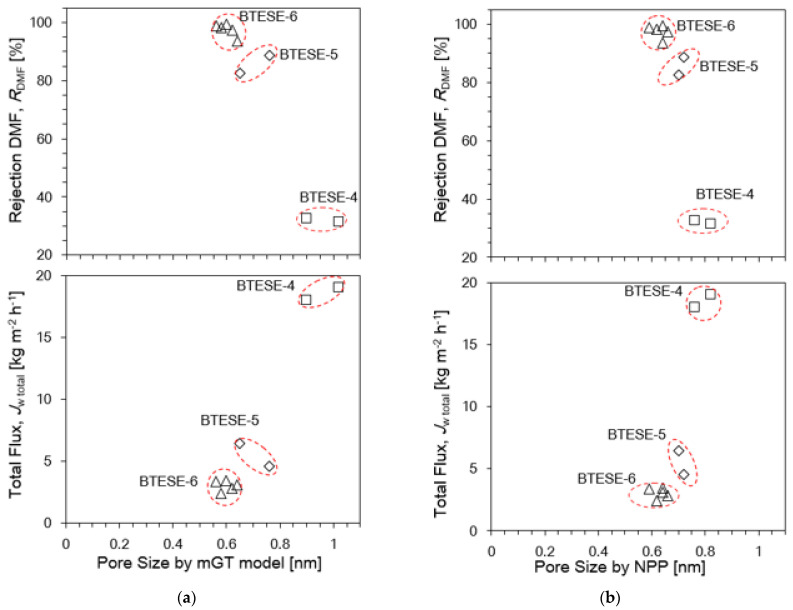
(**a**) Rejection DMF, *R*_DMF_ and Total Flux, *J*_w,total_ versus Pore Size by mGT model. (**b**) Rejection DMF, *R*_DMF_ and Total Flux, *J*_w,total_ versus Pore Size by NPP for BTESE organosilica membranes fired at 300 °C under air as a function. *P*_f_: 8 MPa; at *T*_f_: 25 °C; *C*_DMF_: 6 wt.% DMF; *Q*_f_: 30 mL/min.

Based on the above findings, the pore size data from GT and NPP methods will be used to investigate on how the flux and rejection behaves in relation to the pore size. As mentioned earlier, GT and NPP methods can estimate the nominal pore sizes value. The pore sizes for each of the BTESE-4, BTESE-5 and BTESE-6 membranes can be defined approximately around 0.7 to 1.0 nm, 0.6 to 0.8 nm and 0.5 and 0.7 nm, respectively. However, although these three membrane types show the sub-nanopores pore size (<1 nm), Figure 12a,b show that the DMF rejection and flux pattern for these three membranes differ significantly.

BTESE-4 membranes show a low rejection of DMF but high fluxes with pore size around 0.7 to 1 nm. This can be attributed to the existence of large or defect pores, as there is a constant relative permeance > 6 nm in the NPP plot shown in Figure 4a. This implies that the DMF can pass through these larger/defect pores.

**Figure 13:**
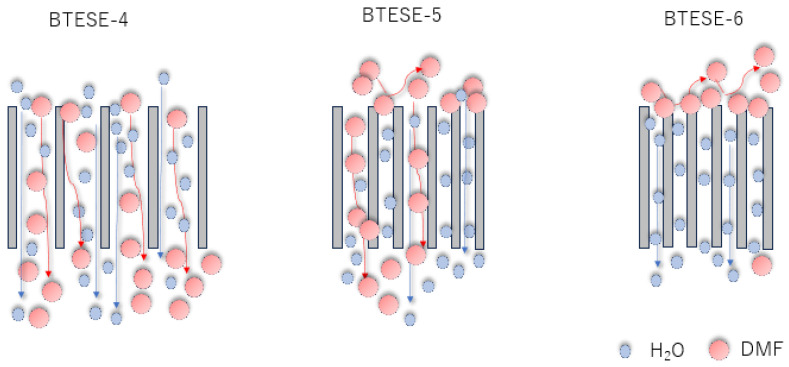
DMF and Water permeation through different pores of the BTESE-4, BTESE-5 and BTESE-6 membranes.

As for the BTESE-5 membrane, this membrane shows a moderate DMF rejection and fluxes despite a considerable portion of defects pores. If the defect pores are reduced to a minimum, the performance of the RO membranes is mostly determined by the nominal pore size as exhibited by BTESE-6 membrane. BTESE-6 membranes show a very good pore size distribution with a mean pore size of 0.5 to 0.7 nm and no big pores larger than 2 nm. It can be seen that there is a significantly high DMF rejection (>95%) but the reduction of the pore size entails a drastic decline in permeate fluxes. All these results clearly show that if the high RO performance membrane is required, the reduction of the number of defect pores in the separation layer is essential. Hence, increasing the coating layer helps to reduce the formation of the defect pores and tune the pore size. Figure 13 illustrates the schematic permeation pattern of the solute and solvent throughout the pore sizes of the different types of membranes.

A selection of BTESE membranes utilized in RO and PV applications are displayed in Table 3. BTESE membranes were previously used in RO application [11,43] to separate aqueous solutions of sodium chloride (NaCl) at low feed pressures of about 1 MPa and temperature variations between 25 and 80 °C. On the other hand, Xu et al. [44] used bis(triethoxysiyl)ethylene (BTESEthy) and modified BTESEthy membranes to separate the same concentration of the aqueous sodium chloride (NaCl) at an operating condition of 1.15 MPa and 25 °C. Only Dong et al. [30,45] discussed the use of bis(triethoxysiyl)acetylene (BTESA) membranes for high feed pressure (6–12 MPa) RO in the separation of methanol (MeOH) from toluene (TOL), methanol (MeOH) from methyl acetate (MA), methanol (MeOH) from dimethyl carbonate (DMC), and methanol (MeOH) from methyl tert-butyl ether (MTBE). The total flux, *J*_w,total_ ranges from 7 to 19 kg/m^2^ h, which indirectly raises the possibility of separating organic solvent mixtures by using organosilica membranes. All these three materials, BTESE, BTESEhy and BTESA, had similar bridge lengths with two carbon atoms, and the difference between these three materials was the degree of saturation of each bond (single, double and triple bond of each organic bridge) [46]. In addition, BTESE membranes also had been widely used in PV and VP for H_2_O/alcohols (e.g., EtOH, IPA, Butanol (BuOH) [47,48,49,50], H_2_O/NaCl [51], H_2_O/Acetic Acid (AA) [52], H_2_O/Ethyl Acetate (EA) [53] and H_2_O/Acetone (Ace) [54] separations. It should be noted that Dong et al. directly compared the separation performance of PV and RO using the same membranes. Higher flux and separation factors were obtained via PV due to a high chemical potential difference [30], which is confirmed using the generalized solution diffusion model. They also theoretically calculated energy requirement for RO, PV and distillation, and reported that RO uses less than one tenth the energy of PV. This suggests that RO can be used to save energy [30].

As mentioned previously, there is limited research on the H_2_O/DMF separation using the RO system. Hence, we compared the *J*_w,total_ and separation factor of the membranes of this work with other membranes used in PV [2,3,31,32,33,34] as a process to separate H_2_O/DMF solution. The direct comparison between RO and PV is difficult, since the phases of feed and permeates of RO and PV are liquid/liquid and liquid/vapor, respectively, and the driving forces are quite different with each other. In addition, PV and RO are generally used in high and low DMF concentrations, respectively, and operated at different temperatures and pressures. Figure 13 shows separation factors as a function of total fluxes, and the detailed operating condition can be found in Table 4. Rejection was converted to separation factor using Equation (17). It should be noted that, when the same membranes are used for RO, PV always shows higher flux and α than RO due to the higher driving force as reported experimentally and theoretically [44].

(17) $$\alpha =\frac{C_{p\;H2O}/C_{pDMF}}{C_{fH2O}/C_{fDMF}}$$

**Figure 14:**
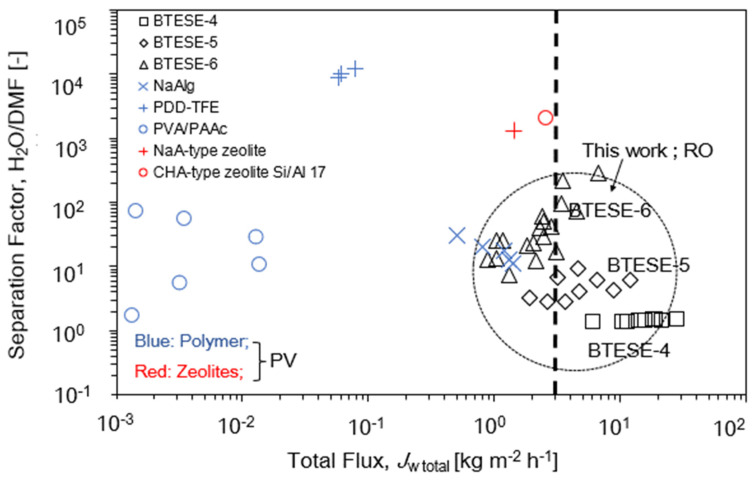
Trade-off between separation factor H_2_O/DMF and total flux, *J*_w,total_ for different membrane materials.

As shown in Figure 14, Solak et al. [2,3] found that the permeation flux of the NaAlg membrane increased while the separation factor showed the vice versa performance with the increasing H_2_O concentration on the feed side (from 20 to 80 wt.%) at an operating temperature of 40 °C. Meanwhile, John and Kamalesh [3,31] proved that their per-fluoro-2,2-dimethyl-1,1,3-dioxole copolymerized with tetrafluoroethylene (PDD-TFE) polymer membrane exhibits high separation H_2_O/DMF~1000 to 10,000 at H_2_O feed concentrations around 10 wt.%; however, the flux can be considered low as the value is below 0.1 kg m^−2^ h^−1^ although the PV was operated at a temperature of 50 °C. On the other hand, Hasegawa et al. [32,33] show that two types of zeolites, NaA and CHA, exhibited a separation factor ~1000–2000 with fluxes around 1.5 to 3 kg m^−2^ h^−1^. In this study, they applied a temperature of around 75 °C to separate H_2_O/DMF. Zhang et al. [34] in their study also show that with an increase in the operating temperature from 25 to 50 °C, the permeation flux of the PVA/PAAc membranes increased, but the separation factor decreased. As we compared the performance of our BTESE membranes with other membrane materials [2,3,31,34], our BTESE membranes can still be considered high-performance membranes in terms of fluxes and *R*_DMF_. The *J*_w,total_ value of our BTESE membranes exhibits higher fluxes more than 3 kg m^−2^ h^−1^ compared to the other membrane materials that used PV as their separation process. The most interesting finding is that our small-pore-size membrane, BTESE-6, with high *R*_DMF_ > 95% (RO), still exhibits high flux in the range of 3–6 kg m^−2^h^−1^ with a separation factor of H_2_O/DMF in the range of 80–120, although the high temperature that we used in our study is only 50 °C.

## 4. Conclusions

The present study marks the successful preparation of the 40 cm long BTESE membranes having a different pore size to be used as an organic aqueous reverse osmosis membrane (RO) in separating of H_2_O/DMF. By adjusting the number of coating layers, the permeation properties of these membranes were enhanced. At a higher number of coating layers, the smaller pore network membrane (BTESE-6) is formed; hence, it can be seen that there is a higher H_2_/SF_6_ (>3000) and DMF rejection (>95%) for this membrane. The result is clear that there was consistency between the average pore sizes found by mGT and those found by nanopermporometry. Furthermore, the correlation between gas and liquid permeance was examined through the utilization of He gas as a water permeance predictor and SF_6_ gas as a DMF permeance predictor. Both the rejection of DMF and the permeance of water increased as the He permeance ratio increased. More intriguingly, when the performance was compared with other PV membranes for H_2_O/DMF separation, our BTESE-6 membranes still showed high flux in the range of 3–6 kg m^−2^h^−1^ with a separation factor H_2_O/DMF in the range of 80–120, demonstrating unequivocally that organosilica membranes are promising for the separation/concentration of organic aqueous solutions.

## Figures and Tables

**Table 1:** List of membranes sample.

Membrane Lists	Coating Times of Top Layers	BTESE Sols for Top Layers
BTESE-4-1	4	1 wt.% BTESE sol after aged for 8 days at 50 °C
BTESE-4-2		
BTESE-5-1	5	
BTESE-5-2		
BTESE-6-1	6	
BTESE-6-2		
BTESE-6-3		
BTESE-6-4		
BTESE-6-5		

**Table 2:** Summary of gas permeance and selectivity at 200 °C for BTESE membranes.

Membrane Numbers	He Permeance (10^−6^ mol m^−2^ s^−1^ Pa^−1^)	Permeance Ratio (-)	
		He/N_2_	He/SF_6_
BTESE-4-1	5.6	4.0	25
BTESE-4-2	7.0	3.3	23
BTESE-5-1	3.5	4.0	54
BTESE-5-2	2.8	5.5	153
BTESE-6-1	3.7	5.0	1968
BTESE-6-2	3.4	4.6	260
BTESE-6-3	2.8	6.3	691
BTESE-6-4	2.3	9.0	3406
BTESE-6-5	2.9	7.1	2940

**Table 3:** RO and PV performance of organosilica membranes (* RO: Feed Pressure; ** PV: Permeate Pressure).

Membrane Code	Separation Method	Feed/ Permeate Pressure	Temperature (°C)	Types of Chemicals Separation	Total Flux, *J*_w,total_ (kg m^−2^ h^−1^)	Solutes Rejection, (%)	Separation Factor, (-)	Ref.
BTESE-4	RO	4–8 MPa *	25–50	6wt.% DMF/ 94 wt.% H_2_O	6–28	25–32	1–2	[This work]
BTESE-5	RO	4–8 MPa *	25–50	6wt.% DMF/ 94 wt.% H_2_O	3–12	63–89	3–9	[This work]
BTESE-6	RO	4–8 MPa *	25–50	6wt.% DMF/ 94 wt.% H_2_O	1–7	86–99	25–285	[This work]
BTESE WR 3	RO	1 MPa *	25–80	2wt.% NaCl/ 98 wt.% H_2_O	0.5–2.8	94–97	1700–3401	[11]
BTESE WR 3	RO	1 MPa *	25–80	0.5wt.% EtOH/ 99.5 wt.% H_2_O	1–3.2	49 25	197 133	[11]
BTESE WR 3	RO	1 MPa *	25–80	0.5wt.% IPA/ 99.5 wt.% H_2_O	0.7–3.3	79 55	478 223	[11]
BTESE WR 240	RO	1 MPa *	25–80	2wt.% NaCl/ 98 wt.% H_2_O	0.1–0.3	94 98	1700 5100	[11]
BTESE WR 240	RO	1 MPa *	25–80	0.5wt.% EtOH/99.5 wt.% H_2_O	0.07–0.4	90 70	1000 335	[11]
BTESE WR 240	RO	1 MPa *	25–80	0.5 wt.% IPA/ 99.5 wt.% H_2_O	0.06–0.4	96 92	2500 1200	[11]
BTESE/ PEG10	RO	1.2 MPa *	25	2 wt.% NaCl/ 98 wt.% H_2_O	0.72	97	34	[43]
BTESE/ PEG10	RO	1.2 MPa *	25	10 wt.% NaCl/ 90 wt.% H_2_O	0.43	95	22	[43]
BTESEthy	RO	1.15 MPa	25	2 wt.% NaCl/ 98 wt.% H_2_O	0.74	97		[44]
BTESEthy-MSA	RO	1.15 MPa	25	2 wt.% NaCl/ 98 wt.% H_2_O	1.23	98		[44]
BTESA	RO	2–8 MPa *	50	5 wt.% TOL/ 95 wt.% MeOH	0.25–2	94–98	1700–500	[30]
BTESA	RO	8–14 MPa *	50	55 wt.% TOL/ 45 wt.% MeOH	0.1–0.9	76–93	900–3000	[30]
BTESA	RO	6–12 MPa *	50	95 wt.% MA/ 5 wt.% MeOH	8.5–19	75–85	7900–13,000	[45]
BTESA	RO	6–12 MPa *	50	95 wt.% DMC/ 5 wt.% MeOH	7.5–17.5	85–89	13,000–18,000	[45]
BTESA	RO	6–12 MPa *	50	95 wt.% MTBE/ 5 wt.% MeOH	7–15.5	94–96	33,000–49,000	[45]
BTESE	PV	<1 kPa **	70	95 wt.% EtOH/ 5 wt.% H_2_O	0.73	-	156	[47]
BTESE	PV	<1 kPa **	55	95 wt.% MeOH/ 5 wt.% H_2_O	0.27	-	4	[47]
BTESE	PV	<1 kPa **	85	95 wt.% IPA/ 5 wt.% H_2_O	1.76	-	3700	[47]
BTESE	PV	<1 kPa **	95	95 wt.% BuOH/ 5 wt.% H_2_O	2.33	-	4700	[47]
BTESE	PV	<1 kPa **	95	95 wt.% BuOH/ 5 wt.% H_2_O	3.3	-	2600	[48]
BTESE/ RTES	PV	<1 kPa **	90	2 wt.% BuOH/ 90 wt.% H_2_O	1.2–1.5	-	15	[49]
BTESE/ RTES	PV	<1 kPa **	60	2 wt.% BuOH/ 90 wt.% H_2_O	0.5–0.6	-	15	[49]
BTESE/ TEOS	PV	<1 kPa **	75	60 wt.% IPA/ 40 wt.% H_2_O	14	-	300	[50]
BTESE/ TEOS	PV	<1kPa	75	90 wt.% IPA/ 10 wt.% H_2_O	9	-	900	[50]
BTESE-600	PV	<1 kPa **	70	2 wt.% NaCl/ 98 wt.% H_2_O	13	-	10,000	[51]
BTESE	PV	<1 kPa **	80	90 wt.% AA/ 10 wt.% H_2_O	2.47	-	350	[52]
BTESE	PV	<1 kPa **	80	90 wt.% AA/ 10 wt.% H_2_O	2.07	-	780	[52]
BTESE-M3	PV	<1 kPa **	60	98 wt.% AA/ 2 wt.% H_2_O	0.84	-	>10,000	[53]
BTESE-M3	PV	<1 kPa **	60	95 wt.% AA/ 5 wt.% H_2_O	1.20	-	>10,000	[53]
BTESE hybrid silica	PV	<1 kPa **	45	90 wt.% Ace/ 10 wt.% H_2_O	1.37	-	52	[54]

**Table 4:** Comparison of parameters of different membrane types for H_2_O/DMF separation (* RO: Feed Pressure/** PV: Permeate Pressure).

Membrane Types	Membrane Code	Separation Method	Feed/ Permeate Pressure	Temperature (°C)	H_2_O in Feed (wt.%)	Total Flux, *J*_w,total_ (kg m^−2^ h^−1^)	DMF Rejection, (%)	Separation Factor, H_2_O/DMF (-)	Ref.
Inorganic	BTESE-4	RO	4–8 MPa *	25–50	94	6–28	25–32	1–2	[This work]
Inorganic	BTESE-5	RO	4–8 MPa *	25–50	94	3–12	63–89	3–9	[This work]
Inorganic	BTESE-6	RO	4–8 MPa *	25–50	94	1–7	86–99	25–285	[This work]
Polymer	NaAlg	PV	<1 kPa **	40	80	1.2	-	18	[2]
Polymer	NaAlg	PV	<1 kPa **	40	60	0.8	-	20	[2]
Polymer	NaAlg	PV	<1 kPa **	40	20	0.5	-	30	[2]
Polymer	NaAlg	PV	<1 kPa **	45	80	1.3	-	13	[2]
Polymer	NaAlg	PV	<1 kPa **	50	80	1.4	-	11	[2]
Polymer	PDD-TFE	PV	<1 kPa **	30	10	0.058	-	9000	[31]
Polymer	PDD-TFE	PV	<1 kPa **	50	10	0.078	-	12,200	[31]
Polymer	PDD-TFE	PV	<1 kPa **	60	10	0.060	-	10,500	[31]
Inorganic	NaA	PV	<1 kPa **	75	10	1.45	-	1290	[3,32]
Inorganic	CHA	PV	<1 kPa **	75	10	2.6	-	2000	[3,33]
Polymer	PVA/ PAAc	PV	<1 kPa **	25	17	0.0006	-	3.6	[34]
Polymer	PVA/ PAAc	PV	<1 kPa **	50	17	0.00133	-	1.7	[34]
Polymer	PVA/PAAc-NaA 20%	PV	<1 kPa **	25	17	0.00142	-	73	[34]
Polymer	PVA/PAAc-NaA 20%	PV	<1 kPa **	50	17	0.00346	-	54.6	[34]
Polymer	PVA/PAAc-SBA15%	PV	<1 kPa **	50	17	0.00320	-	5.4	[34]
Polymer	PVA/PAAc-SiO_2_20%	PV	<1 kPa **	50	35	0.01368	-	10.9	[34]
Polymer	PVA/PAAc-SiO_2_-NH_2_20%	PV	<1 kPa **	50	5	0.01294	-	28	[34]

## Data Availability

The data are not publicly available due to ongoing research using a part of the data.
